# New distributional data on *Oxysternon pteroderum* Nevinson, 1892 (Scarabaeidae, Scarabaeinae,Phanaeini) and its possible implications in conservation

**DOI:** 10.3897/zookeys.174.2659

**Published:** 2012-03-09

**Authors:** Filipe M. França, Fernando A. B. Silva, João Gabriel M. Souza, Paschoal C. Grossi, Fernando Z. Vaz-de-Mello

**Affiliations:** 1 Departamento de Biologia, Setor de Ecologia – Programa de Pós-Graduação em Ecologia Aplicada, Universidade Federal de Lavras, Lavras, MG, Caixa Postal 3037, CEP 37200-000, Brazil; 2 Departamento Entomologia – Programa de Pós-Graduação em Entomologia Agrícola, Universidade Federal de Lavras, Lavras, MG, Caixa Postal 3037, CEP 37200-000, Brazil; 3 Laboratório Biologia da Conservação, Universidade Estadual de Montes Claros - UNIMONTES, Montes Claros, MG, Campus Darcy Ribeiro, CEP 39401-089, Brazil; 4 Universidade Federal do Paraná, Departamento de Zoologia, Caixa Postal 19007, CEP 81531-980, Curitiba, Paraná, Brazil; 5 Departamento de Biologia e Zoologia, Universidade Federal de Mato Grosso, CEP 78060–900, Cuiabá, MT, Brazil

**Keywords:** Atlantic Forest, Cerrado, Coleoptera, dung beetles

## Abstract

New distributional data are reported on the rare phanaeine dung beetle, *Oxysternon pteroderum* Nevinson, 1892, based on five specimens recently collected between 1985 and 2010. Before the present study, *Oxysternun pteroderum* had been reported solely from the super-moist Atlantic coastal forests of southeastern Brazil. We now believe that the distribution of *Oxysternun pteroderum* follows the riparian areas of large rivers connected to the super-moist forest ecosystem.

## Abstract (es)

Novos dados de distribuição da espécie *Oxysternon pteroderum* Nevinson, 1892 baseado em cinco espécimes recentemente coletados entre 1985 e 2010. Antes do presente estudo, *Oxysternun pteroderum* tinha sido reportado apenas para Mata Atlântica de áreas costeiras do sudeste do Brasil. Agora, acreditamos que a distribuição de *Oxysternun pteroderum* segue áreas de florestas ripárias de grandes rios que são conectadas com este ecossistema.

## Introduction

*Oxysternon pteroderum* Nevinson, 1892 was described based on specimens from Montevideo (Uruguay), Rio de Janeiro and Espírito Santo (Brazil) (Nevinson 1892). Since then, it has been considered a rare and endemic species from the Brazilian Atlantic forest (Arnaud 2002; Edmonds and Zídek 2004). This speciesis isolated taxonomically and geographically from the other members of the genus and is not easily confused with any congener. The form of the male pronotum and absence in both sexes of tubercles and other protuberances on the head are unique in the genus. Arnaud, 2002, placed *Oxysternun pteroderum* in a subgenus of its own - *Pteroxysternon* Arnaud, 2002. However, regardless of being in the same subgenus or not, those species included by Edmonds in *Mioxysternon* Edmonds, 1972 appear to be more closely related between them than to species in the nominotypical subgenus.

Until now, this species was known only from super-moist Atlantic coastal forests of southeastern Brazil, having been collected in the states of Espírito Santo, Rio de Janeiro and Minas Gerais (in this last case on the Espírito Santo border) (Arnaud 2002; Edmonds and Zídek 2004). As the Atlantic coastal forest is a greatly threatened habitat and the last known record for this species was from 1955, there was good reason to consider *Oxysternun pteroderum* as a species seriously endangered (Edmonds and Zídek 2004). The purpose of this paper is to present new data on recently (post-1955) collected specimens known to us. We are aware of five specimens from different locations in three different states, collected between 1985 and 2010. We comment on these below.

1. The oldest specimen known to us that was collected after 1955 is a green male collected by students of the São Francisco Xavier School in Ipatinga, Minas Gerais, in November 1985. The collection method is unknown. Ipatinga is located on the margin of the Rio Doce, the most important river in this region. The basin of this river is located in southeastern Brazil and has a drainage area of 83,400 km², of which 86 % belongs to the state of Minas Gerais and 14 % to the State of Espírito Santo. The source of this river is located in the mountainous regions of Minas Gerais in the ranges of Mantiqueira and Espinhaço, and its waters travel around 853 km to reach the Atlantic Ocean near the city of Regência, in Espírito Santo. Thus, the gallery forests that follow the rivers in this basin have an important role connecting Atlantic forest in the coastal lowlands with other areas of Atlantic forest at higher altitudes inland. This specimen is housed in the Everardo and Paschoal Grossi private collection in Nova Friburgo, Brazil (EPGC).

2. A green female, deposited at the Canadian Museum of Nature, Ottawa, Canada, was collected using a flight intercept trap (FIT) in an area of lowland primary Atlantic forest in Linhares, Espírito Santo state (BRAZIL, Espírito Santo, Linhares, Fazenda do Macuco, 27-I-2000, 19°03'50"S, 39°58'43"W, 10m, F. Génier & S. Ide legs). Linhares is at the mouth of the Rio Doce mentioned above, and lowland evergreen forest is continuous between this and the site previously mentioned.

3. A second specimen from the Grossi collection is a bluish-green male specimen, collected manually in a tunnel about three centimeters underneath cow dung, on a rural road near the city of Encruzilhada, State of Bahia (BRAZIL, Bahia, Encruzilhada, 12-XII-2007, 15°28'28"S, 40°50'17"W, Grossi, Rafael & Parizotto legs). This road was bordered on one side (about 5 m from the road) with secondary Atlantic forest (Ombrophilous forest), that was very dry and low in height. The opposite side of the road was covered with introduced pasture. This region, in the south of the Bahia, consists of a triple border between the major biomes of Atlantic forest, Cerrado (Brazilian savanna) and Caatinga, which presents a high number of endemic species. The Atlantic forest of this region is also called “Mata Seca" because of its physiognomy and the plant species found there. This is one of the poorest regions in Brazil where forests are being removed for carbon production in the steel factories of Minas Gerais State.

4. One blue male was collected using pitfall traps baited with human feces in a Cerrado (Brazilian savannah) area (BRAZIL: Minas Gerais, Januária, Área de Proteção Ambiental (APA) de Pandeiros, XI-2008, 15°30.487'S, 44°45.614'W, 544 m, J.G.M. Souza leg.) in November, 2008, and is deposited at the Entomology Section of the Zoology collection of Universidade Federal de Mato Grosso, Cuiabá, Brazil. This is the first record of this species in a Brazilian savannah area, increasing greatly our knowledge of the distribution of this species, which was previously only recorded in Atlantic coastal forests (Edmonds and Zídek 2004). However, the place where this individual was collected is about 30 km from the edge of San Francisco River. Both the source and the mouth of this river are located in Atlantic forest regions.

5. Another male with a bluish-green pronotum and completely blue elytra was collected with pitfall traps baited with human feces in a fragment of secondary Atlantic forest in Minas Gerais (BRAZIL: Minas Gerais, Santa Bárbara, Estação de Preservação e Desenvolvimento Ambiental de Peti (EPDA-Peti forest), 01/15-X-2010, 19°53'01.58"S, 43°22'07.41"W, 685 m, F. França & C. Alves leg.) in October, 2010. This location is in the mountains of the Espinhaço Range. Although the typical vegetation of this region is also Atlantic forest, this is an important record because the elevation where the specimen was collected casts doubt on previously assumed geographic restrictions to low altitude Atlantic forest. The Espinhaço Range comprises a group of mountains between the limits 20°35'S and 11°11'S, ranging from the Ouro Branco Mountains, south of the city of Ouro Preto in Minas Gerais to Bahia, where it receives the name of “Chapada Diamantina" (Giulietti et al. 1997). Formed by old intermittent uplifting, from the Paleozoic, this is an important geographical barrier that limits the Atlantic Forest to the eastern mountain range and the Cerrado to western portion.

**Figure 1.**
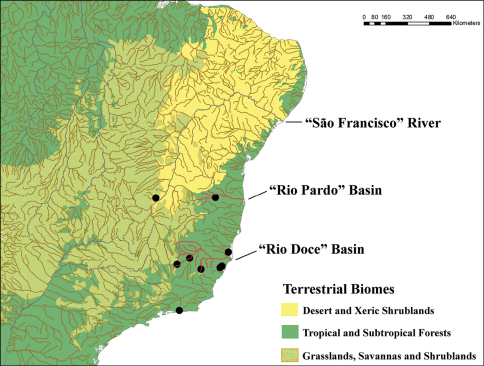
Known geographical distribution of *Oxysternon pteroderum*.

Edmonds and Zídek (2004) reported four localities for this species in the Atlantic forest near the southeastern Brazilian coast (Espírito Santo: Timbuhy, Santa Leopoldina; Minas Gerais: Rio José Pedro; Rio de Janeiro: Rio de Janeiro), in addition to the location of the type-series (Uruguay: Montevideo). With the new records included in this work, we extend the distribution of *Oxysternon pteroderum* to five locations, one being the first record for the state of Bahia (Figure 1). The locations are shown on the map of Figure 1, in which we highlight the following aspects: 1 – points located in the municipalities of Timbuhy (ES), Santa Leopoldina (ES), Linhares (ES) and Rio de Janeiro (RJ) are located in Atlantic forest of low elevations, confirming the previously highlighted relationship of this species with these ecosystems; 2 – the points in the municipalities of Ipatinga (MG), Santa Bárbara (MG), Rio José Pedro (MG), Pandeiros (MG) and Encruzilhada (BA) are at locations away from the Atlantic coast, with some found in Cerrado, or in mountainous regions in southeastern Brazil. However, these points have in common the proximity of major river courses or basins that are connected with the Atlantic forest, such as the Rio Doce (Ipatinga, Santa Bárbara and Rio José Pedro), the basin of the Rio Pardo (Encruzilhada) and the São Francisco River (Pandeiros) (Figure 1). In general, it is common to record dung beetles species typical of Atlantic Forest and Amazonian rainforest along riparian areas that have connections to these ecosystems (Vaz-de-Mello and Silva, personal communication). In addition, the other species included by Edmonds and Zídek (2004) in *Mioxysternon* also present disjunct distribution areas that are connected by large rivers. The distribution limits of these species appear to be relatively wide within a given biogeographic subregion.

We believe the distribution of *Oxysternun pteroderum* follows the riparian areas of large rivers that are connected with this ecosystem. Therefore, we regard the savanna record to be consistent with the assumption that this is a typical species of the Atlantic forest. However, the supposed occurrence of this species in Uruguay (Montevideo), in an area considerably to the south of other known locations, can be a case of incorrect locality record, as already pointed out in the original description of this species (Nevinson 1892).

A large number of pitfalls and/or intercept flight traps were used for sampling specimens in all cases in which *Oxysternun pteroderum* was recorded in this work. However, in all studies, only one individual was recorded. François Génier and Sérgio Ide collected one individual at intercept flight traps and none in several pitfalls baited with human feces installed in the same habitat, at the same time. Grossi and collaborators collected one individual underneath cow dung and none in pitfalls baited with human feces. On the other hand, Souza, França and collaborators collected one individual in pitfalls baited with human feces. The information presented above is not enough to define the food habit of *Oxysternun pteroderum*. Thus, the low abundance observed in collections may be an artefact due to an unknown feeding behavior. The baits traditionally used for collections might not be attractive for this species. However, the hypothesis that *Oxysternun pteroderum* can be a rare species should not be discarded.

This species has not been officially evaluated according to IUCN criteria. Edmonds and Zídek (2004) suggested that *Oxysternun pteroderum* might be endangered due to its highly threatened habitat. The possibility of *Oxysternun pteroderum* inhabiting inland regions of Atlantic Forest through the gallery forests of great rivers decreases its vulnerability because inland regions suffer relatively lower impacts than the coastal areas of this ecosystem. However, we evaluated informally this species using the IUCN criteria and found that it could receive a threatened status mainly due to high fragmentation and destruction of the Atlantic Forest and due to the low number of individuals collected during more than 100 years (we calculated there are less than 35 specimens in collections, including these new records). However, we believe there are still insufficient data to confidently qualify its level of threat due to the poor knowledge of its biology.
